# Peripheral Defocus in Orthokeratology Myopia Correction: Systematic Review and Meta-Analysis

**DOI:** 10.3390/jcm14030662

**Published:** 2025-01-21

**Authors:** António Queirós, Inês Pinheiro, Paulo Fernandes

**Affiliations:** 1 Clinical and Experimental Optometry Research Lab (CEORLab), School of Science, University of Minho, 4710-057 Braga, Portugal; pg50956@alunos.uminho.pt (I.P.); pfernandes@fisica.uminho.pt (P.F.); 2 Physics Center of Minho and Porto Universities, CF-UM-UP, 4710-057 Braga, Portugal

**Keywords:** peripheral refraction, orthokeratology, myopic defocus, myopia progression

## Abstract

**Background:** This study aimed to assess the effect of peripheral defocus with orthokeratology lenses (PDOK) on myopia control in children and adolescents through a systematic review and meta-analysis. **Methods:** A comprehensive search was conducted in the PubMed and Web of Science databases to identify randomized controlled trials (RCTs) and cohort studies on PDOK, using the keywords “peripheral refraction” and “orthokeratology”. Studies were included if they reported spherical equivalent (M) peripheral refraction at 25° and/or 30° with accompanying statistical data along the horizontal meridian before and after orthokeratology treatment. From the initial 133 studies, those excluded included nine non-English publications, 18 reviews, five meta-analyses, four systematic reviews, and 88 studies not meeting the inclusion criteria. **Results:** Nine studies (three RCTs and six cohort studies) were included, involving 259 participants aged six to 30 years with a baseline refractive error of M = −2.44 ± 0.27 D, and treatment duration ranging from 14 days to 12 months. All the studies showed an increase in myopic defocus at 30° nasal (−2.55 ± 1.10 D) and temporal (−2.79 ± 0.75 D) eccentricities, averaging −2.67 ± 0.95 D across both. The overall induced myopic defocus was M = −2.56 D (95% CI: −2.21 to −2.91, Z = 14.33, *p* < 0.001), according to forest plot analysis. Studies with treatment durations up to one year showed a higher myopic blur (M = −2.69 D, 95% CI: −2.48 to −2.89, Z = 25.93, *p* < 0.001) compared to shorter treatments of less than three months (M = −2.39 D, 95% CI: −1.76 to −3.02, Z = 7.41, *p* < 0.001). **Conclusions:** Orthokeratology lenses effectively induce myopic defocus at 30° eccentricity over both short- and long-term treatments in children and adolescents, suggesting potential benefits for myopia control in these age groups.

## 1. Introduction

If no action is taken, 54% of the European population is predicted to be near-sighted (myopic) by the year 2050 [1]. The increasing prevalence of myopia is becoming an emerging public health issue, with growing demand for healthcare resources, high economic burden, and a reduction in the quality of life of myopic individuals. Myopia is one of the leading causes of visual impairment associated with a continuous eye growth. An earlier onset is a great concern, as the more myopic the individual becomes, the greater the risk of them developing serious complications, such as myopic macular degeneration, cataracts, retinal detachment, glaucoma, or even blindness [2].

The exact mechanisms underlying the onset and development of myopia are not yet fully understood. Research suggests that both genetic and environmental risk factors play significant roles. Key environmental contributors include increased time spent indoors, prolonged engagement in visual-near work activities, and extensive use of digital devices. These factors saw a marked rise during the COVID-19 pandemic [3], and its associated home confinement measures, which have been linked to an increase in the progression, prevalence, and incidence of myopia [4].

Initial studies using animal models, particularly with chickens and primates, have provided insights into how altering peripheral refractive error can influence eye growth. When myopic defocus is introduced in the peripheral retina, it appears to signal the eye to slow its axial elongation. Axial elongation is the primary cause of axial myopia, where the eye grows too long, causing light to focus in front of the retina when viewing distant objects. In these animal studies, the induction of a myopic relative peripheral refractive error means that, while the central vision might be emmetropic (focused on the retina), the peripheral regions experience myopic defocus. This myopic defocus in the peripheral retina has been shown to act as a signal to slow down or inhibit axial elongation of the eye [5,6,7]. In contrast, refractive myopia refers to myopia caused by factors other than axial elongation, such as abnormalities in the curvature of the cornea or lens. Since refractive myopia is less influenced by changes in the axial length of the eye, interventions targeting peripheral refractive error may be less effective in these cases [8].

As reported by Fedtke et al. [9], in the first review on peripheral refraction, in 1971 Hoogerheide established the first link between peripheral refraction and myopia in humans. He found that 77% of young emmetropic pilots with hyperopic peripheral refraction developed myopia during their training [10]. Several studies have since reported different patterns of peripheral refraction among different refractive errors. These studies indicate that myopes exhibit hyperopic peripheral defocus, which is associated with axial elongation, while the opposite is observed in emmetropes and hyperopes [11,12,13,14].

Mutti et al., in a 10-year longitudinal study, demonstrated that children who developed myopia showed more relative peripheral hyperopic defocus compared to emmetropic children, two years prior the onset of myopia [15]. Furthermore, Mutti et al. also identified relative peripheral hyperopia as a risk factor for the onset of myopia, independent of central refractive error, although they did not attribute significant importance to this factor in their overall analysis of myopia development [16].

Many therapies for myopia management based on peripheral defocus techniques have emerged and are currently used clinically. These techniques leverage the concept of inducing peripheral defocus to counteract the axial elongation associated with myopia, including progressive, perifocal, and new spectacle lenses with peripheral microlens incorporated, overnight orthokeratology, and peripheral defocus soft contact lenses, including soft multifocal and dual-focus contact lenses. These various techniques have demonstrated differing levels of efficacy and safety in controlling myopia progression [17].

The foundation of orthokeratology for myopia management is predicated on defocusing theory. In contrast to conventional monocular spectacles, which may exacerbate peripheral hyperopia defocusing, orthokeratology modifies the corneal central configuration, facilitating the migration of corneal epithelial cells, mitigating hyperopia defocusing, and delivering myopia defocusing to the peripheral retina via the mechanical pressure exerted by the flat base arc designed in inverse geometry, along with the negative pressure generated by the tear film beneath the reverse arc [18]. Charman et al. showed for the first time that the changes induced on the anterior surface of the cornea by orthokeratology lenses altered the peripheral refraction profile [19]. Queirós et al. later validated these findings and concluded that the amount of myopia induced by orthokeratology treatment, at 30° in the peripheral retina, was directly related to the initial myopia value [20,21].

The primary objective of this study was to quantify the change in peripheral refraction achieved with orthokeratology and to assess the consistency of this outcome across different studies. To the best of the authors’ knowledge, no previous systematic review and meta-analysis has been conducted to address this question.

## 2. Materials and Methods

### 2.1. Search Strategy and Study Selection

This systematic review was conducted in accordance with the Preferred Reporting Items for Systematic Reviews and Meta-Analyses (PRISMA) guidelines [22]. A total of 133 articles, published before 31 May 2024, were identified through searches on the PubMed, Web of Science, and Embase databases. The data search strategy employed Boolean operators and was formulated as follows: “Peripheral Refraction AND Orthokeratology”. The references of the retrieved articles were also reviewed to identify other related studies that met the inclusion criteria. All the included studies had declared ethical approvals and informed consent in the original publications; thus, no ethical approval or informed consent was required for this study.

### 2.2. Study Selection

**Figure 1:**
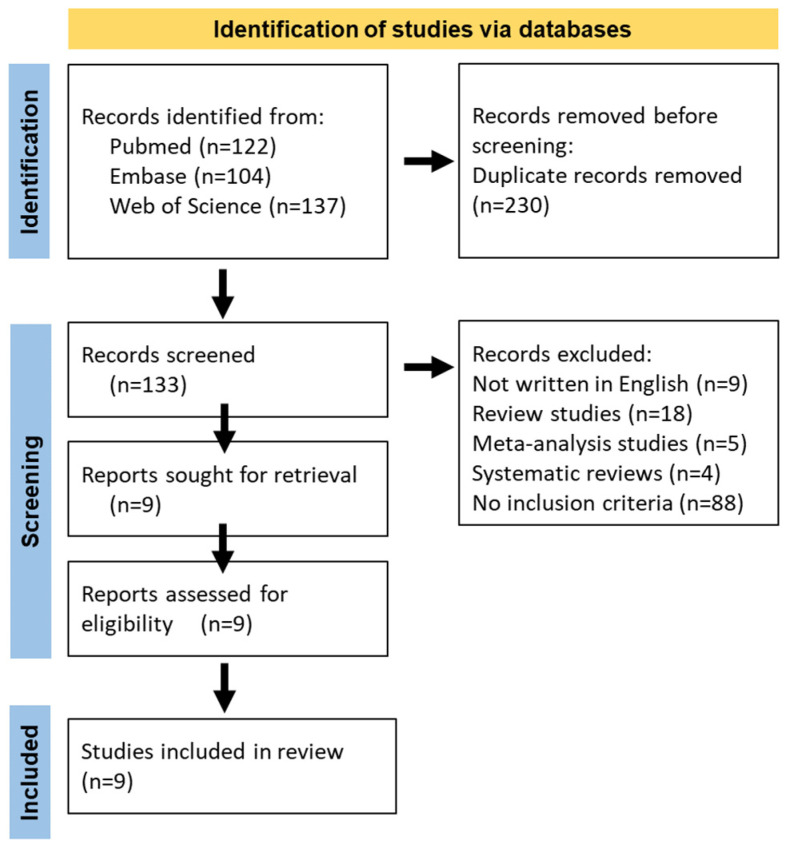
Flowchart of the literature search.

The search strategies for the databases are detailed in Figure 1. Additionally, the references of the included articles were also screened for further eligible studies. The inclusion criteria were as follows: (1) Measurement of peripheral refraction at 25° and/or 30°; (2) provision of standard deviation or standard error of the mean; (3) data from the horizontal meridian; and (4) graphical or tabular presentation of values before and after the intervention. The exclusion criteria included: (1) data to be analyzed could not be extracted or calculated; (2) case reports or series, reviews, comments, editorials, and animal studies; and (3) non-English reports.

### 2.3. Data Extraction and Quality Assessments

Two investigators (AQP and AP) independently screened the titles and abstracts of the selected articles according to the inclusion criteria. A full-text evaluation of the studies was then conducted to determine final eligibility. Any disagreements were addressed through discussion. Furthermore, the following information from the included studies was extracted or calculated from the raw data provided in the articles: last name of the first author, year of publication, country of participants, number of participants, and the aforementioned operational outcomes.

### 2.4. Statistical Analysis

The statistical analysis for the meta-analysis was conducted using Review Manager 5 (RevMan 5, Version: 5.0.25, computer program, Copenhagen: The Nordic Cochrane Centre, The Cochrane Collaboration). This software was utilized to combine the results from individual studies and to calculate pooled effect estimates. Effect measures such as mean differences (MD) with 95% confidence intervals (CIs) were used. Heterogeneity among the included studies was assessed using the I^2^ statistic and the Chi-square test. An I^2^ value greater than 50% and a *p*-value less than 0.05 in the Chi-square test were considered indicative of substantial heterogeneity. In the presence of substantial heterogeneity, a random-effects model was used to account for variability between studies; otherwise, a fixed-effect model was applied. The pooled results were presented in forest plots, showing individual and overall effect sizes with corresponding 95% confidence intervals. This methodological approach ensured a rigorous and comprehensive synthesis of the available evidence, providing reliable estimates of the effects of the interventions studied. For papers that did not present tables of data on peripheral refraction values before and after orthokeratology treatment, the WebPlotDigitizer software (v4) was used to extract values from the figures. At least 3 extractions and comparisons were carried out from the highest resolution figures to ensure the consistency of the retrieved data points. A *p*-value < 0.05 was considered to be statistically significant.

## 3. Results

Table 1 details the nine studies included, according to the defined criteria. These studies involved orthokeratology treatment in 259 eyes, with longitudinal follow-up ranging from 14 days to 12 months. Except for two studies with a shorter duration, all studies were conducted in children aged 6 to 16 years, with a mean age of 14.96 ± 6.54 years and an initial refractive error of −2.44 ± 0.27 D. All studies reported an increase in myopia at 30° nasal (−2.55 ± 1.10 D) and 30° temporal (−2.79 ± 0.75 D).

**Figure 2:**
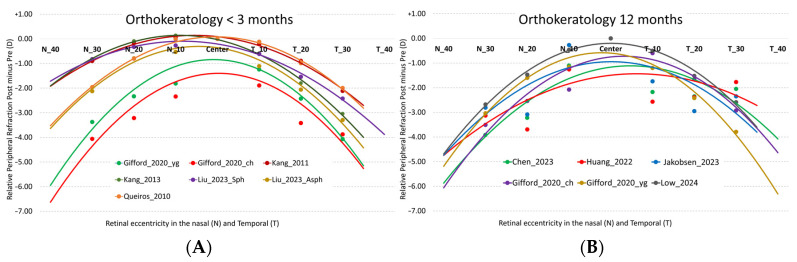
Relative differences (post- minus pre-treatment) in the M component of refraction as a function of field angle in orthokeratology studies in the temporal (T) and nasal (N) retinal area for treatments of less than 3 months (**A**) and 12 months (**B**). The lines represent the second-order polynomial fit for M in the different studies analyzed. Studies under 3 months: Gifford_2020_ch [23], Gifford_2020_yg [23], Kang_2011 [24], Kang_2013 [25], Liu_2023_Asph [26], Liu_2023_Sph [26], Queirós_2010 [21]. 12-months studies: Chen_2023 [27], Gifford_2020_ch [23], Gifford_2020_yg [23], Huang_2022 [28], Jakobsen_2023 [29], Low_2024 [30].

Figure 2 illustrates the relative refractive changes compared to the pre-treatment refractive profile (post- minus pre-treatment) as a function of the study duration. The data represent relative refractive values where the entire curve has been shifted to set the central refractive value to “zero”. This adjustment enhances the visibility of relative changes in peripheral refraction compared to central measurement. The studies analyzed in Table 1 show that orthokeratology induces, on average, a relative peripheral spherical equivalent of −2.67 D ± 0.95 D at 30° nasal/temporal (N/T), −1.91 ± 1.01 D at 20° N/T, and −0.99 ± 0.79 D at 10° N/T. However, when comparing the treatments at 12 months to those at 3 months, it is observed that the myopic shift stabilizes more at 30° N/T (−2.88 ± 0.63 D at 12 months vs. −2.49 ± 1.12 D up to 3 months), with a variation of only 15%. On the other hand, there is greater longitudinal variation for the more central eccentricities (10° N/T with −1.26 ± 0.71 D vs. −0.75 ± 0.77 D and 20° N/T with −2.41 ± 0.70 D vs. −1.49 ± 1.05 D). As a result, from 3 to 12 months at 10° and 20° N/T, a greater myopic increase with treatment is observed, specifically 67% and 61%, respectively.

**Figure 3:**
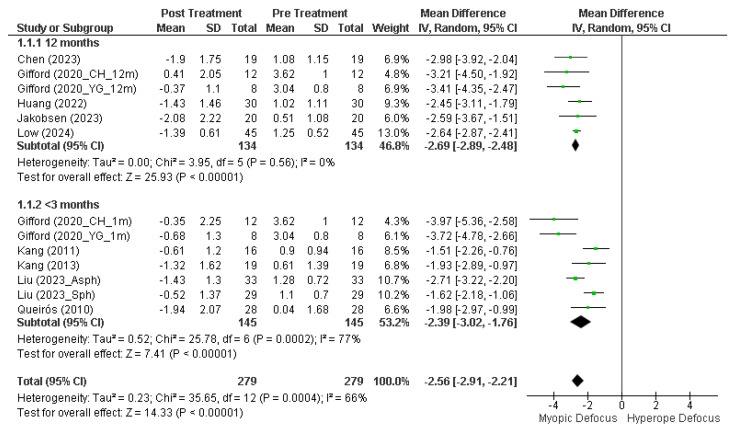
Forest plot of the mean M value at 30° (mean nasal and temporal) after and before orthokeratology treatment. Studies under 3 months: Gifford (2020_CH_1m) [23], Gifford (2020_YH_1m) [23], Kang (2011) [24], Kang (2013) [25], Liu (2023_Asph) [26], Liu (2023_Sph) [26], Queirós (2010) [21]. 12-months studies: Chen (2023) [27], Gifford (2020_CH_12m) [23], Gifford (2020_YG_12m) [23], Huang (2022) [28], Jakobsen (2023) [29], Low (2024) [30].

Figure 3 displays the results of the multiple studies analyzed, divided by longitudinal durations of greater than and less than 3 months. Analysis of the five studies with 12-month duration shows no variability among the studies (Tau^2^ = 0.00) and very low heterogeneity (Chi^2^ = 3.95, *p* = 0.56, I^2^ = 0%). The overall effect size (Z = 25.93, *p* < 0.001) indicates a highly significant effect across all combined studies, with an average myopic defocus of −2.69 D [95% CI, −2.89 D, −2.48 D] induced by orthokeratology treatment.

However, studies with durations of less than 3 months exhibit greater substantial heterogeneity in the results (Chi^2^ = 25.78, *p* < 0.001, I^2^ = 77%), although the effect size remains significant (Z = 7.41, *p* < 0.001). Overall, orthokeratology induces a mean myopic defocus at 30° nasal/temporal of −2.56 D [95% CI, −2.91 D, −2.21 D] (Z = 14.33, *p* < 0.001) in longitudinal terms (Chi^2^ = 35.65, *p* < 0.001, I^2^ = 66%). Despite this substantial heterogeneity, the overall effect is highly significant, indicating that, despite differences among the studies, there is a consistent effect of induction of myopic defocus with orthokeratology treatment.

**Table 1:** Study characteristics of orthokeratology treatment.

Author, Year, Journal	Study Type, Time	Methodology	Country (City), n	Age Mean ± SD (Min, Max, Years)	M_Baseline (Min, Max, (D)
Chen [27], 2023, Frontiers	Prospectively designed, self-controlled observational study, 12 months	Open-field autorefractor WAM-5500 (GrandSeiko Co., Ltd., Hiroshima, Japan) Cycloplegic autorefraction 30 N to 30 T, 5° increment; eye rotate	China (Chengdu), 19	9.84 ± 1.64 (8 to 14)	−2.73 ± 1.09 (−1.00 to −5.00)
Gifford [23], 2020, Contact Lens and Anterior Eye	Prospective, From 1 month to 12 months	Open-field autorefractor Shin-Nippon SRW-5000 (Rexxam Co., Ltd., Osaka, Japan) Non-Cycloplegic autorefraction 30 N to 30 T, 10° increment; eye rotate	Australia (Queensland), 8	13.2 ± 2.1 (8 to 16)	−2.55 ± 1.32 (−0.75 to −5.00)
			Australia (Queensland), 11	23.4 ± 3.5 (19 to 29)	−2.19 ± 0.96 (−1.00 to −3.25)
Huang [28], 2022, GACEO	Prospective, nonrandomized, controlled study, 12 months	Open-field autorefractor WAM-5500 (GrandSeiko Co., Ltd., Hiroshima, Japan) Cycloplegic autorefraction 30 N to 30 T, 10° increment; eye rotate	China (Wenzhou), 30	9.90 ± 1.27 (8 to 13)	−2.63 ± 0.71 (−1.00 to −5.00)
Jakobsen [29], 2023, Acta Ophthal.	Randomized controlled clinical trial, 12 months	Open-field autorefractor Shin-Nippon Nvision-K 5001 (Rexxam Co., Ltd., Osaka, Japan.) Cycloplegic autorefraction 30 N to 30 T, 10° increment; eye rotate	Scandinavian (Danish), 20	9.96 ± 1.54 (6 to 12)	−2.10 ± 1.16 (−0.50 to −4.75)
Kang [24], 2011, OVS	Randomly fitted, 3 months	Open-field autorefractor Shin-Nippon N-Vision K5001 autorefractor (Rexxam Co., Ltd., Osaka, Japan) Non-cycloplegic autorefraction 35 N to 35 T, 10° increment; X	East Asian, 16	x (11 to 16)	−2.37 ± 1.10 (−1.00 to −4.00)
Kang [25], 2013, OPO	Randomly fitted, 14 days	Open-field autorefractor Shin-Nippon NVision-K 5001 autorefractor (Rexxam Co., Ltd., Osaka, Japan) Non-cycloplegic autorefraction 30 N to 30 T, 10° increment, and 35°N,T; X	East Asian, 17	24.2 (18 to 38)	−2.33 ± 1.15 (−1.00 to −4.00)
Liu [26], 2023, CLAE	Randomized, controlled single-masked clinical trial, 3 months	Open-field autorefractor WAM-5500 (GrandSeiko Co., Ltd., Hiroshima, Japan) Cycloplegic autorefraction 30 N to 30 T, 10° increment; X	China (Chengdu), 33	9.43 ± 1.94 (8 to 12)	−2.65 ± 0.80 (−0.75 to −4.00)
			China (Chengdu), 29	9.62 ± 1.08 (8 to 12)	−2.55 ± 0.90 (−0.75 to −4.00)
Low [30], 2024, Clin Optom	Cross sectional study, 12 months	Open-field autorefractor WAM-5500 (GrandSeiko Co., Ltd., Hiroshima, Japan) Cycloplegic autorefraction 30 N to 30 T, 10° increment; eye rotate	Malaysia (Kuala Lumpur), 45	8.38 ± 0.49 (8 to 9)	−2.92 ± 1.07 (−0.75 to −4.00)
Queirós [21], 2010, OVS	Nonrandomized, controlled study, 1 month	Open-field autorefractor WAM-5500 (GrandSeiko Co., Ltd., Hiroshima, Japan) Non-cycloplegic autorefraction 35 N to 35 T, 10° increment; eye rotate	Portugal (Braga), 28	24.6 ± 6.3 (20 to 41)	−1.95 ± 1.27 (−0.88 to −5.25)

## 4. Discussion

To our knowledge, no previous work has analyzed the effect of the peripheral refraction profile at 30° nasal and temporal through a systematic review and meta-analysis in myopic children and adolescents. The results show a significant effect of inducing myopic defocus in all the studies analyzed, regardless of the longitudinal duration of the study. The analysis of the studies included in this meta-analysis showed that the refractive profiles at various retinal eccentricities in all pre-treatments had a hyperopic defocus, whereas after orthokeratology treatment, all studies demonstrated a shift to myopic defocus, with an average difference of −2.56 D at 30°N/T. These findings confirm that orthokeratology consistently induces a myopic refractive profile on the retina within a narrow 95% interval range. However, shorter-term studies present confidence intervals closer to −2.40 D for peripheral defocus.

Several studies showed orthokeratology to be a safe technique [31] with high predictability [32], which is proven to be effective in controlling myopia progression [33] when precise fitting, careful follow-up, and patient compliance with recommended lens cleaning and disinfection are followed [18,34,35]. Peripheral refraction in orthokeratology has been extensively studied, revealing significant findings. Studies have shown that OK induces changes in relative peripheral refraction (RPR) and higher-order aberrations (HOAs) [20,36]. The shift from relative peripheral hyperopia to myopia after OK wear is well-documented, with asymmetrical changes observed in nasal-temporal RPR [27]. After ortho-k treatment, RPR shifts from hyperopia to myopia, particularly in the temporal field, which is associated with slower axial elongation and the nasal-temporal asymmetry in relative peripheral refraction correlated with baseline refractive state. Additionally, children wearing OK lenses have significantly smaller RPRs compared to those wearing single-vision glasses, with a noted correlation between RPR and axial length growth rate [37]. Furthermore, OK wear induces myopic RPR at all eccentricities, with central emmetropization playing a crucial role in optical changes during treatment [29,38]. These findings highlight the importance of understanding peripheral refraction changes induced by OK lenses, providing valuable insights for optimizing myopia management strategies.

The findings of this meta-analysis reinforce prior evidence regarding the effectiveness of orthokeratology in inducing peripheral myopic defocus and confirm that, in the medium and long term, the outcomes across different studies remain consistent and homogeneous. This observed consistency may also account for the uniformity results observed across clinical trials concerning the efficacy of myopia control [39,40,41,42]. Another finding of this meta-analysis (Table 2) is the observed relationship between a greater level of myopic defocus induction and a reduced axial length over 12 months. For instance, Chen et al. (2023) [27] reported a defocus of −2.98 D associated with a 69% control efficacy, while Gifford et al. (2020) [23] documented −3.22 D with 36% efficacy in one cohort and −3.41 D with 24% in another. Similarly, Jakobsen et al. (2023) [29] observed −2.58 D with 46% efficacy, and Low et al. (2024) [30] reported −2.64 D with an 88% reduction in progression. The study by Huang et al. [28], however, represents an exception, with −2.45 D associated with an unexpectedly high efficacy of 112%.

In a recent mini-review, Erdinest et al. highlighted hyperopic peripheral blur as a significant risk factor for myopia development and progression, shedding light on theories related to myopia and axial elongation, with a focus on the impact of peripheral blur on these processes; however, the precise mechanisms linking optical defocus to anatomical eye growth remain unclear [43]. While studies have advanced in identifying potential signals and pathways involved in ocular growth, the relationship between peripheral refraction and refractive error development is not fully understood. Some evidence suggests that the orientation of peripheral blur (horizontal or vertical) serves as an optical cue for the sign of defocus [44], with myopic eyes exhibiting vertically elongated blur and emmetropic or hyperopic eyes showing horizontal elongation. Additionally, ocular aberrations increase with field angle, potentially contributing to differences between refractive groups [45]. Sensitivity to blur decreases with increased retinal eccentricity, influenced by limitations in cone photoreceptors and reduced visual attention, requiring higher levels of defocus in the periphery to elicit a response. However, the precise degree of defocus and the extent of retinal eccentricity that impact foveal refraction remain ambiguous, and the proportion of retinal surface necessary to influence myopia progression remains unclear. Most studies targeting ocular growth focus on the retinal region 20–40 degrees from the visual [43], but further researcher is needed to elucidate the specific defocus parameters most effective for myopia management.

Another important consideration in this meta-analysis is the repeatability and consistency of the results and measurements. All included studies employed the same equipment, ensuring standardization across them. Furthermore, the repeatability of measurements, both intra-visit and inter-visit, has been shown to be high both before and after orthokeratology treatment [46], reinforcing the reliability of the reported outcomes. To assess peripheral refraction the studies included, eye rotation was predominantly used rather than head rotation. This distinction is significant because eye rotation maintains the eye’s optical alignment with the instrument’s measurement axis, potentially reducing measurement variability and improving precision in capturing peripheral refraction. In contrast, head rotation can introduce slight misalignment and may affect peripheral measurements due to changes in ocular torsion [47]. Finally, although the sample predominantly comprised individuals of Asian ethnicity, and minor variations in peripheral refraction measurements may occur due to individual anatomical differences, such as corneal shape, axial length, and retinal contour, particularly across ethnic groups [48], the findings are broadly applicable to the general population. This generalizability is supported by the demonstrated effectiveness of orthokeratology in controlling myopia progression in the Caucasian population as well [33,41,42,49,50,51,52].

Peripheral refraction is crucial in various aspects of visual function and ocular health, and is particularly important in understanding and managing myopia. Studies have shown that induced peripheral refractive errors, such as myopic defocus and/or astigmatism, can reduce visual clarity in the peripheral field and present several changes, particularly in achieving optimal visual acuity and comfort. For instance, they can significantly impair driving performance, which may become more pronounced when drivers engage in secondary tasks, as these tasks can divide attention and make it harder for drivers to compensate for peripheral visual distortions [53,54] Adaptation to the changes in peripheral refraction induced by peripheral defocus, such as multifocal contact lenses or OK, is a critical factor for visual comfort and functional performance [55,56]. Studies have shown that, within about one week of consistent OK lens wear for myopia control, levels often return to baseline levels, suggesting that neural adaptation is capable of overcoming the optical quality degradation induced by the treatment [57]. The visual system undergoes a process where it “learns” to interpret these new signals, reducing perceived visual disturbances [58]. This adaptation likely involves changes in the visual cortex, where neural processing adjusts to compensate for the altered refractive environment and helps maintain visual function and comfort despite changes in optical quality.

Recent research suggests that peripheral refraction may influence the development of myopia based on peripheral lighting conditions. Higher relative peripheral hyperopia is associated with myopic eyes in young adults, particularly under low illuminance conditions [59]. The relationship between peripheral refraction and myopia control has led to the development of advanced technologies to enhance the measurement of peripheral refraction and improve our understanding of its impact on ocular growth [60,61]. Furthermore, investigations into ocular parameters reveal their influence on peripheral ocular refraction, whether through the effect of the crystalline lens [62] or pupil diameter [50]. Changes in the lens curvature or its position can affect how light is refracted at the periphery of the retina. For instance, as the lens accommodates or changes shape, it can alter the peripheral focus, either increasing or decreasing the degree of myopic or hyperopic defocus. This effect is more pronounced in younger individuals, where the lens is more flexible, potentially impacting peripheral refraction during activities that require accommodation [62]. Variations in pupil size significantly affect the amount of light entering the eye through different parts of the lens and cornea, thereby influencing the clarity and distribution of peripheral images. A larger pupil allows more peripheral light rays to reach the retina, which increase optical aberrations and defocus in the peripheral retina. This effect is particularly relevant in OK, where the refractive changes induced by the lenses can become more noticeable under low-light conditions when the pupil dilates. In such scenarios, the increased exposure to peripheral optical distortions may impact the overall visual experience [50].

Comparisons of peripheral refraction in eyes fitted with different myopia control contact lenses have shown variable effects on peripheral refraction [63]. The back optic zone diameter (BOZD) of orthokeratology (OK) lenses notably impacts myopia control, particularly in children. Studies show that OK lenses with a 5 mm BOZD result in lower axial elongation compared to those with larger diameters (6 mm), with reductions of 0.07 mm versus 0.18 mm over one year [64]. The 5 mm BOZD also induced more myopic peripheral refraction, which correlates with reduced axial growth [50,65]. Smaller optical zones create a more pronounced mid-peripheral ring effect [50]. However, larger optical zones may improve visual quality and comfort, suggesting a trade-off between myopia control efficacy and visual performance. This underscores the importance of individualized approaches for effective myopia management [66].

The use of ortho-k over an extended period in myopic children and young adults allows for good visual acuity with rare severe complications like microbial keratitis, making it a safe and effective optical solution [67] and do not compromise the possibility of future LASIK surgery for myopia correction [68]. With OK, there is a higher likelihood of adverse events compared to conventional contact lenses, with a reported odds ratio of 3.79 [69]. Minor complications associated with OK may include issues such as mild corneal staining, transient dry eye, and mild discomfort during initial lens adaptation. While most reported adverse events are relatively mild and reversible, the increased risk of complications is a significant consideration for both practitioners and patients. Education on proper hygiene practices and compliance with treatment are key factors to minimize risks and enhance treatment effectiveness [70,71].

The analysis of the limitations of this study mainly concerns the availability of eligible original resources. Thus, the analysis was restricted to summary results (SE, peripheral refraction at 30°, SD or SEM, horizontal meridian, or graphical presentation), since raw data were not generally provided by the authors. However, the specific indications for conducting the meta-analysis were considered optimal for presenting an overview of the effect of orthokeratology on peripheral refraction in longitudinal terms. In addition, there are intrinsic issues in the studies that can introduce heterogeneity into the analysis, including variations in study designs, follow-up periods, orthokeratology lens profile designs, data presentations, and ethnic, demographic, and other aspects, which may influence this meta-analysis. To address this, we employed a random-effects model, which is generally considered appropriate when combining data from studies with varied designs and follow-up durations. This approach takes into account the variability between studies and provides more conservative pooled estimates, increasing the robustness of the results. In addition, we conducted a subgroup analysis based on follow-up periods (e.g., a shorter period versus longer than three months) to assess any differential effects related to study duration. Also, differences in lens design, such as the base curve and peripheral treatment zone, may lead to variations in how effectively peripheral myopic defocus is induced. Nevertheless, the consistent findings across studies lend support to the observed effect of orthokeratology in inducing peripheral myopic defocus and provide a high level of scientific evidence. However, future studies with a rigorous design covering those different areas are needed to quantify the amount of the induced peripheral myopic defocus to evaluate the effects of orthokeratology in myopia control.

Currently, several clinical approaches can be employed to decelerate myopia progression, all of which have the potential to significantly slow myopia progression with quality of vision largely unaffected and satisfactory safety. This include administering low-dose atropine eye drops or utilizing peripheral defocus therapies such as orthokeratology lenses, soft contact lenses for myopia control, and spectacle lenses with peripheral lenslets [17]. When choosing an appropriate strategy, the risk-to-benefit ratio needs to be weighed up for the individual needs and factors such as age, health, ethnicity, lifestyle, and the rate of myopia progression should be considered.

The research surrounding OK is primarily focused on its efficacy as a treatment for myopic progression in children, for which it can significantly reduce axial elongation over two years. This meta-analysis has highlighted an important area for further research, including the quantification of the peripheral myopic defocus with OK treatment. However, other optical treatments, addressing both axial elongation and myopic defocus, have shown similarly promising results and should also be considered as viable alternatives. The selection of an appropriate treatment demands a thorough analysis of the risk-benefit profile, particularly in pediatric populations, where the role of the practitioner is essential in identifying safe and effective therapeutic options. Comparative studies are crucial to assess not only the efficacy of myopia management strategies but also factors such as safety, treatment adherence, and impact on quality of life. For instance, multifocal contact lenses, which reduce the risk of corneal complications and eliminate the need for night-time handling, may offer significant advantages for specific patient groups. On the other hand, orthokeratology lenses provide unique benefits, such as eliminating the need for optical correction during the day, which can significantly enhance quality of life and improve treatment adherence, particularly for active patients [72]. However, these benefits must be carefully weighed against associated risks, underscoring the importance of rigorous monitoring and comprehensive education on lens hygiene and handling practices. Additionally, recent advances in optical and pharmacological interventions, as well as combined therapies, have expanded the horizons of myopia management. Innovative technologies and an evolving understanding of genetic and environmental factors driving myopia progression pave the way for increasingly integrated and effective approaches [73].

## 5. Conclusions

The results of this study reinforce that orthokeratology is effective in inducing peripheral myopic defocus, regardless of the duration of contact lens wear. This consistent refractive effect may also explain the uniform efficacy of orthokeratology to manage progressive myopia with different lens designs, in different ethnicities and different geographical locations. However, despite its effectiveness in myopia control, the adoption of orthokeratology lenses should involve a careful evaluation of the risk–benefit profile, discussed with parents and taking into account the child’s quality of life, particularly in pediatric populations.

## Figures and Tables

**Table 2:** Effect of axial length changes at 12 months with orthokeratology treatment and the mean value of the relative peripheral refraction difference (post minus pre-treatment) at 30° nasal and temporal, as well as the percentage of myopia progression control of these studies.

Study	Mean RPR @ 30°N/T (D)	Control Efficacy (%)	Effect Size in Axial Length (mm)
Chen_2023 [27]	−2.98 ± 1.32	69%	0.17
Gifford_2020 [23]	−3.22 ± 0.41	−36%	−0.09
Gifford_2020 [23]	−3.41 ± 0.53	−24%	−0.06
Huang_2022 [28]	−2.45 ± 0.96	112%	0.28
Jakobsen_2023 [29]	−2.58 ± 0.33	46%	0.11
Low_2024 [30]	−2.64 ± 0.06	−88%	−0.21
Mean	−2.88 ± 0.63	13%	0.03

## Data Availability

Data supporting the reported results can be requested from the authors.
